# Streptozotocin-induced diabetes in the rat is associated with changes in vaginal hemodynamics, morphology and biochemical markers

**DOI:** 10.1186/1472-6793-6-4

**Published:** 2006-05-30

**Authors:** Noel N Kim, Miljan Stankovic, Tulay T Cushman, Irwin Goldstein, Ricardo Munarriz, Abdulmaged M Traish

**Affiliations:** 1 Institute for Sexual Medicine, Department of Urology, Boston University School of Medicine, Boston, USA; 2 Department of Anatomy and Neurobiology, Boston University School of Medicine, Boston, USA; 3 Department of Biochemistry, Boston University School of Medicine, Boston, USA

## Abstract

**Background:**

Diabetes is associated with declining sexual function in women. However, the effects of diabetes on genital tissue structure, innervation and function remains poorly characterized. In control and streptozotocin-treated female rats, we investigated the effects of diabetes on vaginal blood flow, tissue morphology, and expression of arginase I, endothelial nitric oxide synthase (eNOS) and cGMP-dependent protein kinase (PKG), key enzymes that regulate smooth muscle relaxation. We further related these changes with estrogen receptor alpha (ERα) and androgen receptor (AR) expression.

**Results:**

In addition to significantly elevated blood glucose levels, diabetic rats had decreased mean body weight, lower levels of plasma estradiol, and higher plasma testosterone concentration, compared to age-matched controls. Eight weeks after administration of buffer (control) or 65 mg/kg of streptozotocin (diabetic), the vaginal blood flow response to pelvic nerve stimulation was significantly reduced in diabetic rats. Histological examination of vaginal tissue from diabetic animals showed reduced epithelial thickness and atrophy of the muscularis layer. Diabetic animals also had reduced vaginal levels of eNOS and arginase I, but elevated levels of PKG, as assessed by Western blot analyses. These alterations were accompanied by a reduction in both ERα and AR in nuclear extracts of vaginal tissue from diabetic animals.

**Conclusion:**

In ovariectomized (estrogen deficient) animals, previous reports from our lab and others have documented changes in blood flow, tissue structure, ERα, arginase I and eNOS that parallel those observed in diabetic rats. We hypothesize that diabetes may lead to multiple disruptions in sex steroid hormone synthesis, metabolism and action. These pathological events may cause dramatic changes in tissue structure and key enzymes that regulate cell growth and smooth muscle contractility, ultimately affecting the genital response during sexual arousal.

## Background

Diabetes has adverse effects on sexual function in women [1-3]. Symptoms consistent with autonomic neuropathy have been associated with decreased subjective sexual arousal in diabetic women [4]. In a recent study, Erol *et al*. noted that women with type II diabetes experienced higher prevalence rates of sexual dysfunction when compared to non-diabetic patients [5]. Approximately 80% of diabetic patients complained of loss of libido, 60% had diminished clitoral sensitivity, 50% experienced orgasmic dysfunction, 40% experienced vaginal discomfort, and 40% experienced vaginal dryness. Similarly, other clinical studies have demonstrated that women with type I diabetes had a higher prevalence of sexual dysfunction (27%) than healthy women of similar age (15%) [2]. Type I diabetic women experienced higher prevalence of reduced vasocongestion and reduced vaginal lubrication to erotic stimuli [2,6]. Thus, these clinical studies suggest that both type I and type II diabetes impedes the physiological arousal response in women.

The physiology of vaginal hemodynamics and lubrication responses associated with sexual arousal is highly dependent on the integrity of tissue structure and function and involves complex neuro-vascular processes modulated by various local neurotransmitters, vasoactive agents, sex steroid hormones and growth factors [7-15]. During sexual arousal, activation of parasympathetic nerves to the genital organs results in relaxation of the vaginal wall, increased blood flow, vasocongestion (engorgement), and production of plasma transudate in the vagina [16,17]. These events are modulated by the tone of the smooth muscle in both the vasculature and the muscularis. While the neurogenic mechanisms are incompletely characterized, adrenergic and nitrergic nerves have been demonstrated to be important regulators of vascular and non-vascular smooth muscle tone in the vagina [17].

In laboratory studies, vaginal wall contraction to norepinephrine and relaxation to nitric oxide were both attenuated in diabetic animals [8]. Diabetes has also been reported to reduce epithelial growth and decrease blood vessel density in the lamina propria of the rat vagina [18]. These changes were also accompanied by increased tissue fibrosis and elevations in TGF-β1. Thus, laboratory and clinical studies suggest that diabetes-induced alterations in neural and vascular systems may bring about pathophysiological changes that impact negatively on sexual function. However, such studies are few in number and limited in scope.

Accumulating evidence suggests that estradiol may protect against diabetic complications [19-21]. Given the important role of estradiol in the maintenance of vaginal health and function, studying the role of estrogens in diabetes with regard to vaginal physiology provides an interesting point of convergence for the physiology of steroid hormones and the pathophysiology of diabetes. The effects of diabetes on vaginal tissue structure, innervation and function remains poorly characterized and little is known regarding the effects of diabetes on the regulation of expression or function of sex steroid receptors and enzymes that synthesize neurotransmitters (*e.g*. nitric oxide synthase). The objective of this study was to investigate the effects of diabetes on vaginal blood flow, tissue morphology, and expression of key biochemical markers in the animal model that may be important for vaginal vasocongestion and lubrication responses during sexual arousal. Using streptozotocin-treated female rats, a widely used model of type I diabetes, we examined changes in the levels of arginase I, endothelial nitric oxide synthase (eNOS) and cGMP-dependent protein kinase (PKG) due to their roles in modulating smooth muscle relaxation [11-13,22-25]. We further related these changes with estrogen receptor expression and function.

## Results

### Physiological parameters and vaginal blood flow

Administration of streptozotocin resulted in significant elevations (≥ 7-fold) in blood glucose levels after 2 weeks that were sustained throughout the duration of the study (Table 1). On average, rats had a mean body weight of 209 ± 6 g at the beginning of the study. Although diabetic animals on normal diet gained weight after 8 weeks (18% increase), they gained less weight than control animals (35% increase) and had significantly lower body weights when compared to controls (Table 1). Interestingly, while vaginal weight and length were similar for both treatment groups, uteri from diabetic rats were significantly smaller and had lower wet weights relative to control animals. In addition, diabetic animals had a lower concentration of estradiol and a higher concentration of testosterone in plasma samples.

**Table 1:** Blood glucose, body and tissue weights, and plasma hormone concentrations of control and diabetic rats. Female Sprague-Dawley rats, at 8–10 weeks of age, were fasted for 18–24 h and administered 0.02 M citrate saline buffer (Control) or streptozotocin (65 mg/kg) in citrate buffer (Diabetic) by intraperitoneal injection. All parameters shown below were determined 8 weeks after the administration of streptozotocin or buffer. Comparisons between groups were performed using unpaired t-test and two-tailed p values are listed below each parameter examined.

	Blood Glucose (mg/dL)	Body Weight (g)	Uterine Weight (mg)	Vaginal Weight (mg)	Vaginal Length (mm)	Plasma Hormone Conc.	
						
						Estradiol (pg/mL)	Testosterone (pg/mL)
Control (n = 11)	68.7 ± 2.2	281.2 ± 6.1	644.8 ± 55.3	238.1 ± 10.5	20.0 ± 0.9	58.3 ± 4.4	185.8 ± 15.8
Diabetic (n = 12)	497.6 ± 28.0	247.1 ± 8.5	437.4 ± 57.2	237.7 ± 9.5	18.7 ± 0.3	47.3 ± 3.1	281.2 ± 28.4
P (two-tailed)	<0.0001	0.0048	0.0183	0.9776	0.1644	0.0508	0.0146

**Figure 1:**
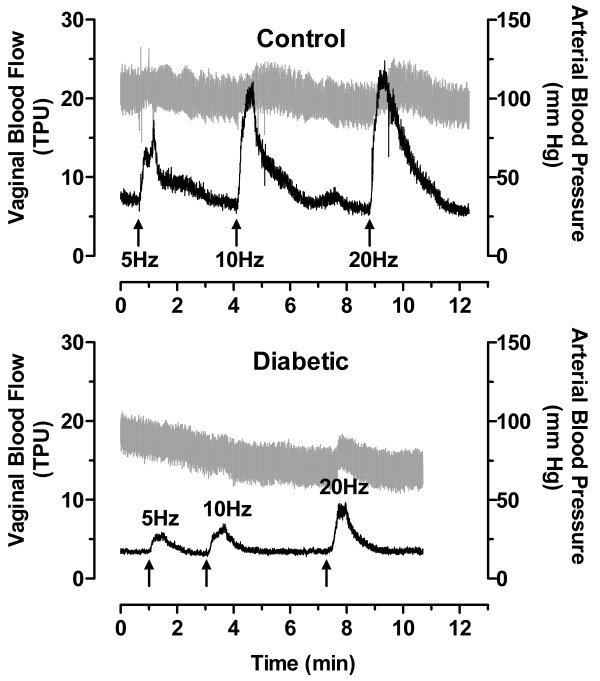
Effect of diabetes on vaginal blood flow in rats. Female Sprague-Dawley rats, at 8–10 weeks of age, were fasted for 18–24 h and administered 0.02 M citrate saline buffer (Control) or streptozotocin (65 mg/kg) in citrate buffer (Diabetic) by intraperitoneal injection. After 8 weeks, rats were anesthetized and the vaginal blood flow response to pelvic nerve stimulation was recorded by laser Doppler flowmetry. The pelvic nerve was stimulated for 30 seconds at 5, 10, and 20 Hz. Shown are representative real time recordings of vaginal blood flow (black) expressed as tissue perfusion units (TPU; left axis) and systemic arterial pressure (gray) expressed as millimeters of Hg (right axis).

**Figure 2:**
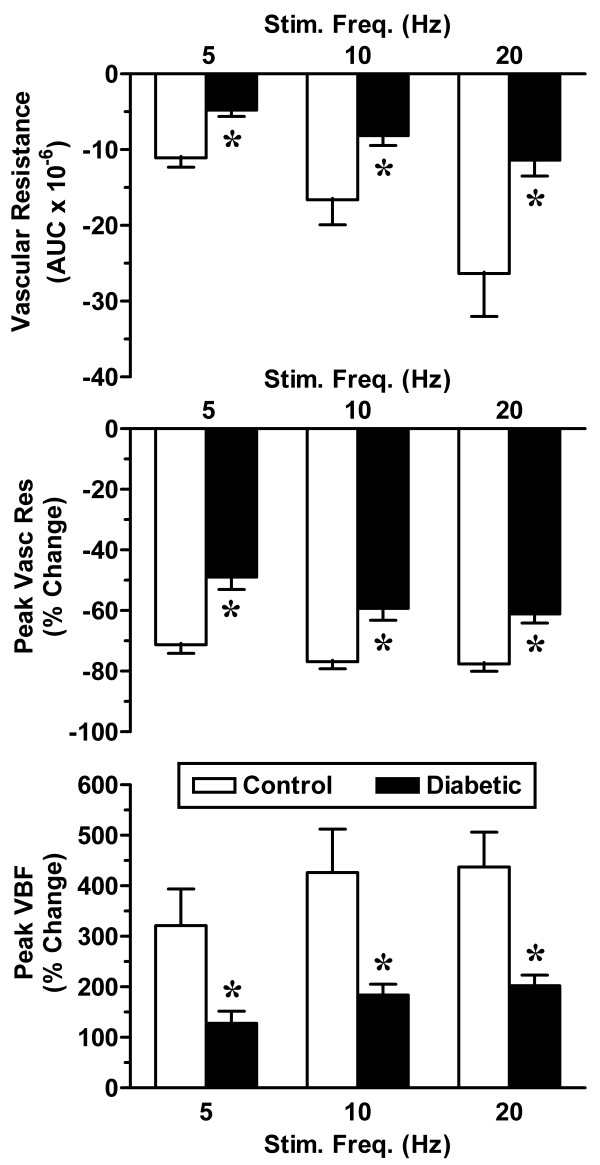
Summarized data for vaginal blood flow. Vaginal blood flow was measured in control (open bars) and diabetic groups (solid bars) of female rats (see Figure 1). Responses to pelvic nerve stimulation were analyzed by calculating the area-under-the-curve (AUC) for vascular resistance (systemic arterial pressure/blood flow) and the percent change in peak vascular resistance and vaginal blood flow (VBF). Data are the mean ± SEM. *p < 0.05 versus control group.

Blood flow in the vagina was assessed by laser Doppler flowmetry. In control rats, vaginal blood flow in response to pelvic nerve stimulation was proportional to the stimulation frequency (Figure 1). However, in diabetic animals, the amplitude of the blood flow response was significantly reduced (Figures 1 &2) and was not always proportional to the frequency of nerve stimulation. In addition, the duration of each nerve-stimulated response tended to be shorter and the basal blood flow in the absence of nerve stimulation was lower in diabetic animals. To factor in the contribution of any changes in systemic blood pressure, we also calculated the vascular resistance in the vagina. Vascular resistance was characteristically decreased in response to stimulation of the pelvic nerve. Whether vascular resistance was assessed by the area under the curve or the average peak response, diabetic animals exhibited a significant attenuation in this parameter of vaginal perfusion (Figure 2).

### Vaginal tissue morphology

**Figure 3:**
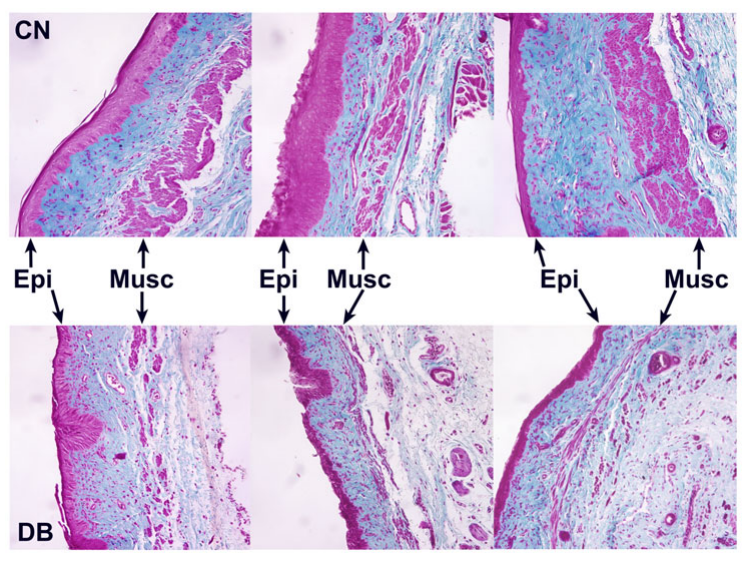
Effect of diabetes on overall vaginal tissue structure. Vaginal tissue sections (5 μm) from control (CN) and diabetic (DB) rats (see Figure 1) were subjected to a modified Masson's trichrome staining procedure. Shown are representative tissue sections from 3 separate animals for each treatment group. For each panel, the epithelium (Epi) and muscularis (Musc) layers are identified by the arrows. Magnification = 240 ×

Although mean vaginal tissue wet weights and lengths were similar in both control and diabetic groups, vaginas from diabetic rats appeared atrophic upon gross examination. To further study potential diabetes-induced changes, vaginal tissue sections were subjected to Masson's trichrome staining. This procedure stains cellular material purple-red and extracellular material (primarily connective tissue) blue-green (Figure 3). In control animals, the thickness of the epithelium varied, depending on the stage of the estrous cycle when the tissue was removed and fixed. Examination of vaginal smears indicated that almost half (5/11) of the control rats were in diestrus, with the remaining animals evenly distributed between proestrus, estrus and metestrus (2/11 for each stage). In all control animals, irrespective of the estrous cycle stage, the muscularis layer consisted of large, well-defined bundles of smooth muscle (Figure 3).

In the diabetic group, 75% of the animals (9/12) were in diestrus, 2 rats were in metestrus, 1 rat was in estrus, and none were in proestrus. Vaginal tissue cross-sections from diabetic rats tended to have epithelium that was more uniformly thin with fewer layers of cells, relative to control animals. Most strikingly, the muscularis layer was consistently thin with less well developed bundles, suggesting atrophy (Figure 3). Alterations to the lamina propria were not evident. However, the use of more specific immunostaining would be required to detect changes in blood vessel structure or density.

### Protein expression of biochemical markers related to vaginal hemodynamics

**Figure 4:**
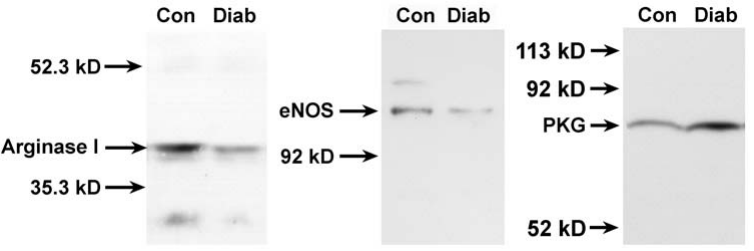
Effect of diabetes on arginase I, eNOS, and PKG protein expression in the rat vagina. Vaginal tissues from diabetic (Diab) or age-matched control (Con) rats were homogenized and the cytosolic fraction was obtained by differential centrifugation. The vaginal tissue cytosolic extract (200μg total protein/lane) was then subjected to gel electrophoresis under denaturing conditions. Gels were blotted onto nitrocellulose membranes that were probed with primary antibodies against arginase I, eNOS and PKG. After incubation with the secondary antibody, membranes were developed using an enhanced chemiluminescence reaction and bands were visualized by exposure to film.

To further characterize potential mechanisms that might be responsible for the decreased vaginal blood flow in diabetic rats, we examined the protein levels of arginase I, endothelial nitric oxide synthase (eNOS), and cGMP-dependent protein kinase (PKG); key enzymes that are known to regulate vascular smooth muscle tone within the vagina. On average, levels of arginase I protein decreased by 57% and eNOS protein decreased by 40% in vaginal tissue from diabetic rats, relative to age-matched controls (Figure 4). However, PKG protein levels in the vagina increased approximately 2-fold in the diabetic group, relative to the control group.

### Expression of estrogen and androgen receptors

**Figure 5:**
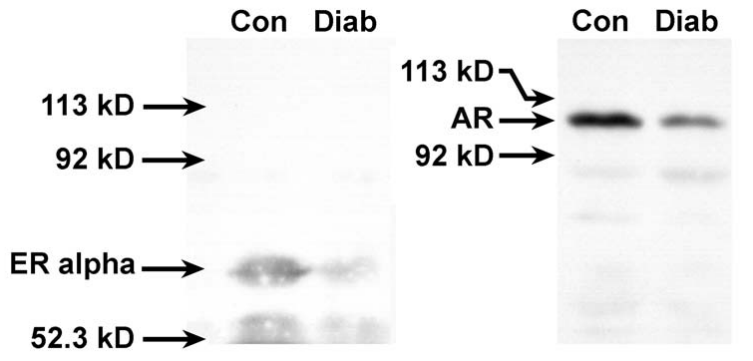
Effect of diabetes on nuclear estrogen receptor alpha (ERα) and androgen receptor (AR) in the rat vagina. Vaginal tissues from diabetic (Diab) or age-matched control (Con) rats were homogenized and the nuclear subcellular fraction was obtained by differential centrifugation. ERα was extracted from the nuclei by homogenization and incubation in high salt buffer. The vaginal tissue nuclear extract (200 μg total protein/lane) was then subjected to gel electrophoresis under denaturing conditions. Gels were blotted onto nitrocellulose membranes that were probed with antibody against ERα or AR. After incubation with the secondary antibody, membranes were developed using an enhanced chemiluminescence reaction and bands were visualized by exposure to film.

In light of the changes in the plasma concentration of estradiol and testosterone in diabetic rats, we investigated the effects of diabetes on the protein levels of estrogen and androgen receptors in the vagina. Nuclear extracts were examined by Western blot analyses to determine levels of hormone-activated receptor. On average, ERα decreased in vaginal tissue from diabetic rats by 51% and AR protein levels decreased by 69% when compared to age-matched controls (Figure 5).

## Discussion

These data suggest that the diabetic state produces dramatic changes in vaginal tissue structure and function, characterized by decreased blood flow, atrophy of the muscularis and attenuation of epithelial proliferation. These changes were also accompanied by alterations in sex steroid hormone receptors and key enzymes that regulate blood flow. Previous studies in the rat demonstrated that preservation of normal vaginal tissue structure and blood flow are dependent upon estrogen signaling [13,26]. On a gross level, the histological and physiological changes in the present study resemble those observed in vaginal tissue from ovariectomized animals in previous studies [13,15,26]. Similar to ovariectomized animals, mean plasma estradiol concentration and mean uterine wet weight was decreased in the diabetic group. However, unlike ovariectomized animals, plasma testosterone levels were significantly elevated in the diabetic group. While the reasons for this increase remain unclear, previous studies have demonstrated that ovarian aromatase activity is insulin-dependent [27-29]. In the STZ-induced diabetic rat, the lack of insulin production may inhibit aromatase activity, causing an increase in testosterone due to decreased estrogen biosynthesis. In addition, female rats repeatedly subjected to physical and psychological stress protocols are reported to have significantly elevated plasma testosterone [30], suggesting that increased testosterone may be part of a systemic stress response.

It remains to be established whether the lower levels of circulating plasma estradiol are responsible for the changes in the diabetic rats. Yet, given that vaginal tissue from diabetic animals exhibited decreased ERα protein in the nucleus, it is likely that additional mechanisms that interfere with estrogen receptor signaling are triggered. This perspective is further supported by the observation that in vaginal tissue of ovariectomized animals, ERα increases in the nuclear compartment but decreases in the cytosolic compartment [13]. If decreased plasma estradiol alone were responsible for the alterations in vaginal tissue structure and function, one would expect similar intracellular distributions of ERα in both diabetic and ovariectomized animals. Since the severity and duration of estrogen deficiency is different in the ovariectomized rat, additional studies are necessary to define the time course of changes in the diabetic rat and the dose-response relationship between estrogen and vaginal structure and function. The decreased levels of nuclear AR in diabetic rat vaginal tissue suggests that testosterone action may also be disrupted in the diabetic state. While testosterone has been shown to affect adrenergic nerve density in the rat vagina [26], the role of androgens in regulating vaginal structure and function in diabetes remains to be examined.

Further parallels between the diabetic and ovariectomized states are highlighted by the reductions in arginase I and eNOS in vaginal tissue. Previous studies have shown that arginase in rabbit vagina [31] and eNOS in rat vagina [32] are decreased in ovariectomized animals and up-regulated after estrogen administration. However, some of these changes appear to be tissue and species specific, since estradiol can have the opposite effect on eNOS expression or activity in rabbits [33,34]. While arginase has been shown to modulate vascular responses in female genital tissue, it has also been shown in other tissues to be critical for regulating cell growth [35,36]. Lower arginase levels may be partially responsible for the atrophic appearance of the vaginal muscularis and epithelium in diabetic animals. In addition, arginase activity can decrease NO synthesis through substrate competition (*i.e*. by decreasing intracellular arginine pools) and the lower levels of arginase in diabetic rat vagina may also be reflective of a compensatory down-regulation in the local vasculature to maintain basal perfusion.

This compensatory response may also involve the observed changes in PKG. Since PKG mediates the intracellular response to NO, decreased NO production and/or increased NO scavenging in diabetic animals could lead to a compensatory up-regulation in PKG. Alternatively, the protein level and enzyme activity of PKG have been reported to increase in the lung following chronic hypoxia [37]. It is possible that decreased blood flow to the vagina in diabetic animals may have produced hypoxic conditions that triggered the up-regulation of PKG. Interestingly, PKG was significantly decreased in penile tissue of diabetic male rabbits [38]. This contrasting finding may be attributed to any number of differences, including tissue, species, sex, duration of diabetes or mechanism of function.

Thus, data from the current study, together with our previous findings, suggest that diabetes may adversely affect estrogen production and/or ER signaling. This hypothesis is derived from several lines of evidence that include parallel changes with regard to blood flow, tissue structure, and protein expression of arginase and eNOS that are observed in both diabetic and ovariectomized (estrogen deficient) animals. The dissimilar changes of nuclear ERα levels in diabetic and ovariectomized animals, and the observation that many diabetic rats are in diestrus (phase of estrous cycle with lowest estrogen level), further support a pathophysiological mechanism that includes disruptions in estrogen action.

Previous laboratory studies have indicated that diabetes disrupts estrogen signaling in a variety of tissues. In the pituitary of diabetic animals, ER binding was reduced and ERα levels decreased, potentially affecting sexual receptivity and reproduction [39,40]. Nuclear retention of estradiol-bound ER has been shown to be of shorter duration in both the pituitary and uteri of diabetic animals [41,42]. Further, pituitary responsiveness to LH-RH was reduced, inhibiting ovulation in diabetic animals [43]. In STZ-induced diabetic mice, estradiol restored cell proliferation in the dentate gyrus and subventricular zone [44] and high glucose has been shown to block the effects of estradiol in human vascular cell growth [45]. Diabetes is also known to adversely affect renal function and estradiol treatment counteracts this pathophysiological effect in diabetic animals [46]. More specific investigation into disease mechanisms affecting ER regulation indicate that oxidative stress, a well-known consequence of diabetes, differentially regulates the expression of ERα and ERβ in different cell types [47]. Thus, the diabetic state may cause dysregulation of estrogen action within both central and peripheral estrogen-sensitive tissues.

It should be noted that our study is a preliminary report and has several important limitations. Histological changes in the vagina were only assessed on a gross level and not analyzed by objective morphometric methods. More detailed studies examining diabetes-induced structural changes in the vagina are currently planned. These studies would quantitatively determine structural changes in the various layers of the vagina, blood vessel density, innervation, etc. Further, since the Western blot assays were performed with whole tissue extracts, the cellular localization of the biochemical markers that were examined in this study remains unclear. Immunohistochemical staining of these markers would provide additional information pertinent to the effects of diabetic impairment of vaginal function. Future histological studies should also examine changes in structure and protein expression in different regions of the vagina. Only tissue sections from the mid-vagina were used in the current study since this is the region where blood flow was measured. Also, tissues from multiple animals in each treatment group were pooled for Western blot analyses. Values reported as percent change (relative to control) were intended to be indicative of general trends and should not be taken as exhaustive quantitative determinations. Finally, vaginal smears were not performed daily to assess potential disruptions in the estrous cycle and the increased number of diabetic rats in diestrus may have been purely coincidental. However, the decrease in plasma estradiol and uterine weight, the general atrophic appearance of the vagina, and the reduced levels of nuclear ERα in vaginal tissue of diabetic animals are consistent observations that are suggestive of disruptions in steroid hormone synthesis and signaling. We did not determine ERβ levels in our study since ERα has been shown to be the predominant subtype expressed in the rat vagina [48].

While the data from this study are limited in their interpretation with respect to the clinical state of diabetes, this model provides data supporting clinical observations of decreased genital arousal in women with diabetes mellitus. If indeed STZ-induced diabetes in the rat disrupts estrogen signaling, then it would be logical to investigate in future studies the role of estrogen therapy in diabetes to ameliorate the complications of diabetes.

## Conclusion

In summary, we hypothesize that STZ-induced diabetes leads to multiple disruptions in sex steroid hormone synthesis, metabolism and action. In the vagina, diabetes causes a significant reduction in blood flow. This pathological alteration in a critical component of the genital response during sexual arousal is accompanied by dramatic changes in tissue structure and key enzymes that regulate cell growth and smooth muscle contractility. Future studies should address these molecular events in greater detail and examine the potential sex specific nature of diabetic manifestations.

## Methods

### Animals

Mature female Sprague-Dawley rats (strain Crl:CD; 8–10 weeks old; 200–225 g) were purchased from Charles River Laboratories (Wilmington, MA) and used for investigation of the effects of diabetes on vaginal physiology. These studies were approved by the Institutional Animal Care and Use Committee at the Boston University School of Medicine. Animals were fasted for 18–24 h and streptozotocin (STZ; 65 mg/kg) in 0.02 M citrate saline buffer was administered intraperitoneally, as described previously [8,18,49]. The age-matched control group received citrate buffer only. Blood glucose levels were monitored every 2 weeks using an *Accu-check *blood glucose meter (Roche Diagnostics, Basel, Switzerland). Rats with blood glucose levels ≥ 15 mM (200 mg/dl) for 2 consecutive weeks were considered diabetic. Eight weeks after the initial administration of streptozotocin or buffer, *in vivo *studies were carried out to assess vaginal blood flow, as described below.

### Assessment of estrus cycle phase

For all intact animals, smears of vaginal cells were prepared before vaginal blood flow measurements. A cotton swab moistened in 0.9% saline was inserted into the vagina, manipulated in a circular fashion and then smeared onto a glass slide. Samples were fixed with 70% ethanol and stained with hematoxylin and CytoQuik stain (Fisher Scientific). The phase of the estrous cycle was determined from the morphological characteristics of the vaginal cells. All samples were coded to mask the treatment groups and evaluations were performed by a licensed cytologist. The actual estrous phase of each animal remained unknown to the investigators until data analysis was complete.

### In vivo assessment of vaginal blood flow

Changes in vaginal blood flow in response to pelvic nerve stimulation were determined by laser Doppler flowmetry, as previously described [12,13]. Briefly, rats were anesthetized with intramuscular injections of ketamine (40 mg/kg) and xylazine (4 mg/kg). To continuously monitor systemic blood pressure, a 24-gauge angiocatheter was introduced into the carotid artery and connected to a pressure channel of the laser Doppler flowmeter (Model BLF21D, Transonic Systems, Inc., Ithaca, NY). A surface probe (Type I; Transonic Systems, Inc.) was placed in the vaginal canal, facing the anterolateral vaginal wall and approximately halfway between the introitus and the cervix. Unilateral pelvic nerve stimulation (PNS) was accomplished with a Grass S9 stimulator set at normal polarity and repeat mode to generate a 30 second train of square waves with 6 Volt pulse amplitude and 0.8 millisecond pulse duration at various frequencies (5, 10, 20 Hz). Blood flow data were recorded as tissue perfusion units (TPU). Hemodynamic parameters (vaginal blood flow and systemic blood pressure) were continuously recorded (collection rate = 100 Hz) throughout the experiment using WinDaq software (version 2.15; Dataq Instruments, Akron, OH). Since blood flow through any given tissue is dependent upon the systemic blood pressure, we also calculated the instantaneous vascular resistance (systemic blood pressure/blood flow) at each time point. Using the vascular resistance plots, the area under the curve (AUC) for each response was determined. The results were expressed as mean ± S.E.M. Comparisons between treatment groups were analyzed by unpaired t-tests. Means were considered significantly different if the two-tailed p-value was less than 0.05.

### Measurement of plasma hormones

Blood was drawn at the end of the vaginal blood flow studies. Plasma samples were sent to the Endocrinology Laboratory, Animal Health Diagnostic Center at the Cornell University College of Veterinary Medicine (Ithaca, NY) for determination of estradiol and testosterone levels. A solid-phase radioimmunoassay (RIA) with Coat-A-Count reagents (Diagnostic Products Corp., Los Angeles, CA) was used, as previously described [50].

### Tissue procurement

After the completion of the *in vivo *studies, animals were exsanguinated and vaginal and uterine tissues were removed in their entirety, weighed and placed in chilled physiological salt solution. For histological analyses, vaginas (5 animals per group) were opened with a longitudinal incision and a small notch was cut at the distal end for proper orientation. Tissues were then immersed in 10% neutral buffered formalin and stored at 4°C for 24–72 hours. For biochemical studies, vaginal tissues from control (n = 6) or diabetic (n = 7) animals were frozen in liquid nitrogen and stored at -80°C until assayed.

### Histology

After fixation, the mid-section of each vagina was embedded in paraffin and subsequently sectioned at 5 microns using a rotary microtome. Tissue sections were deparaffinized with CitriSolv (Fisher Scientific, Pittsburgh, PA), rehydrated in graded ethanol solutions (100–70%), and subjected to a modified Masson's trichrome staining procedure, as previously described [26].

### Preparation of cytosolic and nuclear extracts from vaginal tissue

Frozen vaginal tissue was pulverized with a Bessman tissue pulverizer (Spectrum Laboratories, Rancho Dominguez, CA) cooled on dry ice. Tissue powder from each treatment group was pooled and combined (1 g/4 ml) with ice cold buffer (20 mM HEPES, pH 7.4, 1 mM EDTA, 0.25 M sucrose). After the addition of phenylmethylsulfonylfluoride (PMSF; 0.5 mM) and mammalian protease inhibitor cocktail (1 mM AEBSF, 0.08 μM aprotinin, 20 μM leupeptin, 40 μM bestatin, 15 μM pepstatin A and 14 μM E-64; Sigma Chemical Co., St. Louis, MO), tissue powder was homogenized on ice using a Brinkmann PT3000 polytron with 10 second bursts and 30 second cooling intervals. The homogenate was centrifuged at 3000 × g for 20 min at 4°C and the supernatant (cytosol) was transferred to a fresh tube. An aliquot was used to determine soluble protein concentration and the rest of the cytosolic extract was frozen at -20°C until further assay. The pellet containing the nuclei was resuspended in ice cold buffer (50 mM Tris-HCl, pH 7.4 at 22°C, 1.5 mM sodium EDTA, 10% glycerol, 10 mM sodium molybdate and 10 mM monothioglycerol) containing 0.4 M KCl and homogenized on ice with a Dual glass-glass homogenizer using a motor driven pestle. The nuclear homogenate was incubated on ice for 1 hour and centrifuged at 100,000 × g for 30 minutes. The resulting supernatant (nuclear extract) was transferred to a new tube and frozen at -20°C.

### Western blot analyses

Western blot analyses were performed on cytosolic extracts of vaginal tissue to assess changes in arginase I, endothelial nitric oxide synthase (eNOS), and cGMP-dependent protein kinase (PKG). Nuclear extracts of vaginal tissue were used to assess changes in estrogen receptor alpha (ERα) and androgen receptor (AR). Aliquots of vaginal tissue extract (200 μg/lane) were electrophoresed on 7.5% polyacrylamide/10% SDS gels under denaturing conditions and transferred to nitrocellulose membranes. Membranes were incubated for 1 hr in blocking buffer and then incubated at 4°C for 16–20 hr in primary antibody on a rocking platform. The following primary antibodies were used: rabbit polyclonal anti-arginase I (H-52; Santa Cruz Biotechnology, Inc., Santa Cruz, CA), mouse monoclonal anti-eNOS IgG1 (clone 3; BD Transduction Laboratories, Franklin Lanes, NJ), rabbit polyclonal anti-PKG (Stressgen Biotechnologies, Victoria, BC, Canada), rabbit monoclonal anti-ERα (clone SP1, catalog #RM9101; Lab Vision Corp., Fremont, CA), and rabbit polyclonal anti-AR (PA1-111A; Affinity BioReagents, Golden, CO). All antibodies were used at the dilutions recommended by their respective suppliers. Membranes were washed and incubated with either horseradish peroxidase-linked goat anti-rabbit IgG or goat anti-mouse IgG secondary antibody (Pierce Chemical Co., Rockford, IL). After washing, membranes were developed with an enhanced chemiluminescence (ECL) kit (Pierce Chemical Co.) and exposed to autoradiographic film. Band intensity was quantified with Image J, a public domain Java image processing program [51].

## Authors' contributions

NNK participated in the design of the study, assisted with the animal treatment protocols and data collection, analysed the data, prepared the figures, and maintained a lead role in writing and revising the manuscript. MS generated the control and diabetic animal groups, performed the in vivo blood flow studies, assisted in tissue collection, and participated in revising the manuscript. TTC assisted in tissue collection, carried out all histological processing and evaluations (including estrous cycle staging), and participated in revising the manuscript. IG and RM participated in conceiving the study and assisted with drafting and revising the manuscript. AMT assisted in conceiving and designing the study and participated in drafting and revising the manuscript. All authors have read and approved the final manuscript.

## References

[B1] Enzlin P, Mathieu C, Vanderschueren D, Demyttenaere K (1998). Diabetes mellitus and female sexuality: a review of 25 years' research. Diabet Med.

[B2] Enzlin P, Mathieu C, Van den Bruel A, Bosteels J, Vanderschueren D, Demyttenaere K (2002). Sexual dysfunction in women with type 1 diabetes: a controlled study. Diabetes Care.

[B3] Jovanovic L (2002). Finally, it is our turn!. Diabetes Care.

[B4] Tyrer G, Steel JM, Ewing DJ, Bancroft J, Warner P, Clarke BF (1983). Sexual responsiveness in diabetic women. Diabetologia.

[B5] Erol B, Tefekli A, Ozbey I, Salman F, Dincag N, Kadioglu A, Tellaloglu S (2002). Sexual dysfunction in type II diabetic females: a comparative study. J Sex Marital Ther.

[B6] Wincze JP, Albert A, Bansal S (1993). Sexual arousal in diabetic females: physiological and self-report measures. Arch Sex Behav.

[B7] Giuliano F, Rampin O, Allard J (2002). Neurophysiology and pharmacology of female genital sexual response. J Sex Marital Ther.

[B8] Giraldi A, Persson K, Werkstrom V, Alm P, Wagner G, Andersson KE (2001). Effects of diabetes on neurotransmission in rat vaginal smooth muscle. Int J Impot Res.

[B9] Giraldi A, Alm P, Werkstrom V, Myllymaki L, Wagner G, Andersson KE (2002). Morphological and functional characterization of a rat vaginal smooth muscle sphincter. Int J Impot Res.

[B10] Min K, Munarriz R, Kim NN, Goldstein I, Traish A (2002). Effects of ovariectomy and estrogen and androgen treatment on sildenafil-mediated changes in female genital blood flow and vaginal lubrication in the animal model. Am J Obstet Gynecol.

[B11] Kim SW, Jeong SJ, Munarriz R, Kim NN, Goldstein I, Traish AM (2003). Role of the nitric oxide-cyclic GMP pathway in regulation of vaginal blood flow. Int J Impot Res.

[B12] Kim SW, Jeong SJ, Munarriz R, Kim NN, Goldstein I, Traish AM (2004). An in vivo rat model to investigate female vaginal arousal response. J Urol.

[B13] Kim SW, Kim NN, Jeong SJ, Munarriz R, Goldstein I, Traish AM (2004). Modulation of rat vaginal blood flow and estrogen receptor by estradiol. J Urol.

[B14] Kim NN, Min K, Pessina MA, Munarriz R, Goldstein I, Traish AM (2004). Effects of ovariectomy and steroid hormones on vaginal smooth muscle contractility. Int J Impot Res.

[B15] Park K, Ahn K, Lee S, Ryu S, Park Y, Azadzoi KM (2001). Decreased circulating levels of estrogen alter vaginal and clitoral blood flow and structure in the rabbit. Int J Impot Res.

[B16] Guiliano F, Julia-Guilloteau V, Goldstein I, Meston CM, Davis SR, Traish AM (2006). Neurophysiology of female genital sexual response. Women's Sexual Function and Dysfunction – Study, Diagnosis and Treatment.

[B17] Giraldi A, Levin RJ, Goldstein I, Meston CM, Davis SR, Traish AM (2006). Vascular physiology of female sexual function. Women's Sexual Function and Dysfunction – Study, Diagnosis and Treatment.

[B18] Park K, Ryu SB, Park YI, Ahn K, Lee SN, Nam JH (2001). Diabetes mellitus induces vaginal tissue fibrosis by TGF-beta 1 expression in the rat model. J Sex Marital Ther.

[B19] Louet JF, LeMay C, Mauvais-Jarvis F (2004). Antidiabetic actions of estrogen: insight from human and genetic mouse models. Curr Atheroscler Rep.

[B20] Palin SL, Kumar S, Sturdee DW, Barnett AH (2001). HRT in women with diabetes – review of the effects on glucose and lipid metabolism. Diabetes Res Clin Pract.

[B21] Khoo CL, Perera M (2005). Diabetes and the menopause. J Br Menopause Soc.

[B22] Bredt DS (1999). Endogenous nitric oxide synthesis: biological functions and pathophysiology. Free Radic Res.

[B23] Lincoln TM, Dey N, Sellak H (2001). cGMP-dependent protein kinase signaling mechanisms in smooth muscle: from the regulation of tone to gene expression. J Appl Physiol.

[B24] Sanders DB, Kelley T, Larson D (2000). The role of nitric oxide synthase/nitric oxide in vascular smooth muscle control. Perfusion.

[B25] Kim NN, Christianson DW, Traish AM (2004). Role of arginase in the male and female sexual arousal response. J Nutr.

[B26] Pessina MA, Hoyt RF, Goldstein I, Traish AM (2006). Differential effects of estradiol, progesterone, and testosterone on vaginal structural integrity. Endocrinol.

[B27] Garzo VG, Dorrington JH (1984). Aromatase activity in human granulose cells during follicular development and the modulation by follicle-stimulating hormone and insulin. Am J Obstet Gynecol.

[B28] La Marca A, Morgante G, Paglia T, Ciotta L, Cianci A, De Leo V (1999). Effects of metformin on adrenal steroidogenesis in women with polycystic ovary syndrome. Fertil Steril.

[B29] La Marca A, Egbe TO, Morgante G, Paglia T, Cianci A, De Leo V (2000). Metformin treatment reduces ovarian cytochrome P-450c17α response to human chorionic gonadotrophin in women with insulin resistance-related polycystic ovary syndrome. Hum Reprod.

[B30] Yoon H, Chung WS, Park YY, Cho IH (2005). Effects of stress on female rat sexual function. Int J Impot Res.

[B31] Traish A, Kim NN, Huang YH, Min K, Munarriz R, Goldstein I (2003). Sex steroid hormones differentially regulate nitric oxide synthase and arginase activities in the proximal and distal rabbit vagina. Int J Impot Res.

[B32] Berman JR, McCarthy MM, Kyprianou N (1998). Effect of estrogen withdrawal on nitric oxide synthase expression and apoptosis in the rat vagina. Urology.

[B33] Batra S, Iosif C, Al-Hijji J, Larsson I (2003). Important differences in nitric oxide synthase activity and predominant isoform in reproductive tissues from human and rat. Reprod Biol Endocrinol.

[B34] Yoon HN, Chung WS, Park YY, Shim BS, Han WS, Kwon SW (2001). Effects of estrogen on nitric oxide synthase and histological composition in the rabbit clitoris and vagina. Int J Impot Res.

[B35] Morris SM (2004). Recent advances in arginine metabolism. Curr Opin Clin Nutr Metab Care.

[B36] Li H, Meininger CJ, Kelly KA, Hawker JR, Morris SM, Wu G (2002). Activities of arginase I and II are limiting for endothelial cell proliferation. Am J Physiol.

[B37] Jernigan NL, Walker BR, Resta TC (2003). Pulmonary PKG-1 is upregulated following chronic hypoxia. Am J Physiol.

[B38] Chang S, Hypolite JA, Velez M, Changolkar A, Wein AJ, Chacko S, DiSanto ME (2004). Downregulation of cGMP-dependent protein kinase-1 activity in the corpus cavernosum smooth muscle of diabetic rabbits. Am J Physiol.

[B39] Coirini H, Weisenberg L, Tornello S, De Nicola AF (1980). Effect of experimental diabetes on estradiol binding by the anterior pituitary and hypothalamus in ovariectomized rats. Experientia.

[B40] Siegel LI, Wade GN (1979). Insulin withdrawal impairs sexual receptivity and retention of brain cell nuclear estrogen receptors in diabetic rats. Neuroendocrinology.

[B41] Ekka E, Vanderheyden I, De Hertogh R (1982). Short term nuclear retention and early cytosol replenishment of estradiol receptors in uteri of ovariectomized diabetic rats after intraperitoneal injection of 17 beta-estradiol: evidence for decreased hormonal activity on protein synthesis. Endocrinology.

[B42] Weisenberg L, Fridman O, Libertun C, De Nicola AF (1983). Changes in nuclear translocation of estradiol-receptor complex in anterior pituitary and uterus of rats with streptozotocin diabetes. J Steroid Biochem.

[B43] Blades RA, Bryant KR, Whitehead SA (1985). Feedback effects of steroids and gonadotrophin control in adult rats with streptozotocin-induced diabetes mellitus. Diabetologia.

[B44] Saravia F, Revsin Y, Lux-Lantos V, Beauquis J, Homo-Delarche F, De Nicola AF (2004). Oestradiol restores cell proliferation in dentate gyrus and subventricular zone of streptozotocin-diabetic mice. J Neuroendocrinol.

[B45] Somjen D, Paller CJ, Gayer B, Kohen F, Knoll E, Stern N (2004). High glucose blocks the effects of estradiol on human vascular cell growth: differential interaction with estradiol and raloxifene. J Steroid Biochem Mol Biol.

[B46] Mankhey RW, Bhatti F, Maric C (2005). 17β-estradiol replacement improves renal function and pathology associated with diabetic nephropathy. Am J Physiol.

[B47] Tamir S, Izrael S, Vaya J (2002). The effect of oxidative stress on ERalpha and ERbeta expression. J Steroid Biochem Mol Biol.

[B48] Mowa CN, Iwanaga T (2000). Differential distribution of oestrogen receptor-α and -β mRNAs in the female reproductive organ of rats as revealed by *in situ *hybridization. J Endocrinol.

[B49] Usta MF, Bivalacqua TJ, Yang DY, Ramanitharan A, Sell DR, Viswanathan A, Monnier VM, Hellstrom WJ (2003). The protective effect of aminoguanidine on erectile function in streptozotocin diabetic rats. J Urol.

[B50] Reimers TJ, Lamb SV, Bartlett SA, Matamoros RA, Cowan RG, Engle JS (1991). Effects of hemolysis and storage on quantification of hormones in blood samples from dogs, cattle, and horses. Am J Vet Res.

[B51] Image J: Image processing and analysis in Java. http://rsb.info.nih.gov/ij.

